# Sfrp1 attenuates TAC-induced cardiac dysfunction by inhibiting Wnt signaling pathway- mediated myocardial apoptosis in mice

**DOI:** 10.1186/s12944-018-0832-3

**Published:** 2018-08-28

**Authors:** Shuo Pan, Xiujuan Zhao, Xu Wang, Xin Tian, Yuanbo Wang, Rong Fan, Na Feng, Shumiao Zhang, Xiaoming Gu, Min Jia, Juan Li, Lu Yang, Kaiyan Wang, Haitao Guo, Jianming Pei

**Affiliations:** 1 Department of Physiology, National Key Discipline of Cell Biology, Fourth Military Medical University, Xi’an, Shaanxi Province China; 2 1st Department of Cardiology, People’s Hospital of Shaanxi Province, Xi’an, Shaanxi Province China; 3 Ultrasonic Center, Northwest Women and Children’s Hospital, Xi’an, Shaanxi Province China; 4 Student Brigade, Fourth Military Medical University, Xi’an, Shaanxi Province China

**Keywords:** Heart failure, Wnt signaling pathway, Sfrp1, Viral vector, Apoptosis

## Abstract

**Background:**

Hemodynamic overload causes cardiac hypertrophy leading to heart failure. Wnt signaling pathway was reported activated in heart failure. Secreted frizzled related protein 1 (Sfrp1) is a suppressor of Wnt signaling activation. The aim of the present study was to investigate the protective effect of Sfrp1 on hemodynamic overload- induced cardiac dysfunction.

**Methods:**

A mice transverse aortic constriction (TAC)- induced heart failure model was established. A recombinant adeno-associated virus 9 (AAV9) vector was used to deliver Sfrp1 gene into myocardium. Fluorescence and immunohistochemistry staining was used to evaluate the effectiveness of viral vector delivery. Invasive hemodynamic examination was used to evaluate cardiac systolic and diastolic functions. Myocardium apoptosis was detected by TUNEL assay. The expression levels of Sfrp1, β-catenin, caspase3, Bax, Bcl-2 and c-Myc were measured by Western blotting.

**Results:**

Increased mean arterial pressure and impaired cardiac function confirmed the establishment of TAC model. Sfrp1 protein expression was effectively increased in myocardium of mice treated with AAV9-Sfrp1 viral vector. The viral vector administration improved both systolic and diastolic cardiac functions by reducing myocardial apoptosis in TAC mice. The expression levels of β-catenin, caspase3 and Bax were significantly reduced while the expression levels of Bcl-2 and c-Myc were dramatically increased in myocardium by the viral vector treatment in TAC mice.

**Conclusions:**

AAV9 viral vector delivered sfrp1 expression gene into myocardium, which attenuated TAC-induced cardiac dysfunction by inhibiting Wnt signaling pathway activation- mediated apoptosis.

## Background

Resulted from various pathogens, heart failure has become one of the causes of death leading to heavy public heath burden worldwide [1]. Cardiac remodeling has been identified as a characterized pathological feature of heart failure, which is associated with many cardiovascular diseases such as myocardial infarction and chronic hypertension. Ventricular hypertrophy is indentified as a compensatory process responding to excessive blood pressure overload [2]. It has been established that cardiac hypertrophy is associated with abnormally alterations of structure, metabolism and multiple intracellular signaling transductions [3]. These changes would lead to heart failure when cardiac hypertrophy is decompensated. The involved molecular mechanisms are very complicated and still unclear till now. Thus, deeper investigations are necessary to reveal the mechanisms and significant for finding more therapeutic targets.

One of the critical signaling pathways involved in cardiac hypertrophy is Wingless (Wnt)/β-catenin pathway. Wnt/β-catenin signaling pathway was found playing a critical role in many vital cellular biological processes including cell proliferation, differentiation and migration of many human cell types [4]. Previous studies suggested that β-catenin was a critical regulator of cell proliferation and survival through its targeted genes such as Bcl2 and caspase3 [5]. Among the five kinds of secreted frizzled related proteins (Sfrps), Sfrp1 was proposed to be correlated with cardiac development and several cardiovascular diseases. For instance, it was reported that Sfrp1 was beneficial in recovering cardiac function and structural damage in animal model of myocardial infarction [6]. Sfrp1 was proved to compete the frizzled receptor of Wnt signaling and further to act as a suppressor of Wnt signaling [7].

Based on the above literatures, it was reasonable for us to raise the hypothesis that intentionally Sfrp1 expression enhancement would attenuate the cardiac dysfunction by inhibiting Wnt signaling mediated myocyte apoptosis. In the current study, a mouse model of transverse aortic constriction (TAC)- induced heart failure was established. By using a myocardiotropic viral vector, the Sfrp1 was delivered into animals. The cardioprotective effect of Sfrp1 and the involvement of Wnt signaling were investigated. We believe that results from this study would not only add new information to our current knowledge, but also indicate the possibility of genetic therapy in cardiac hypertrophy.

## Methods

### Animals, TAC procedure and treatments

C57BL/6 mice (8–10 weeks old) were provided by Animal Experiment Center of Xi’an Jiaotong University. Mice were kept in independent cages and were raised in an environment providing a 12-h light/dark circle, 50% humidity and constant temperature at 25 °C. All animals were free to sterile water and standard mice chow. The animal experimental procedures were carried out in accordance to the Recommended Guidelines for Care and Use of Laboratory Animals issued by the Chinese Council on Animal Research. Protocols were reviewed and approved by the Medical Animal Research Ethics Committee of Xi’an Jiaotong University.

The TAC mediated heart failure model was established according to previous descriptions [8]. Briefly, mice were anesthetized by isoflurane inhalation and intubated with a respirator. The chest was opened by a midline sternotomy and the aorta was visualized. A 6.0 prolene suture was placed around the aorta distal to the brachiocephalic artery and then tightened around a 27-gauge needle adjacent to the aorta. The needle was removed after the suture was securely ligated. Then the chest was closed and the animals were continued to be raised for 2 months. Chest was opened without aorta ligation was administrated to mice as sham operations. Several mice were administrated recombinant AAV9 viral vector containing the Sfrp1 gene which was generated and kept in our lab [9]. The viral vectors were delivered to mice at a volume of 100 μL (2 × 10^11^GC/ml) via tail vein injection prior to the animal model establishment.

### Determinations of hemodynamics

The cardiac functions were evaluated with an invasive hemodynamic method as describe previously [10]. After mice were anesthetized by isoflurane inhalation, the right carotid artery was visualized the intubated with a Midro-Tip catheter (Millar) which was connected to a pressure sensor (Omega Bio-Tek). The catheter was inserted into left ventricle which was confirmed by the alteration of pressure curve plotted by the Powerlab 4/25 Biological Analysis system (AD Instruments). The left ventricular systolic pressure (LVSP) and left ventricular end diastolic pressure (LVEDP), mean arterial pressure (MAP) and the maximum rate of left ventricular pressure decay (-dP/dt) were measured and recorded.

### Fluorescence and immunohistochemistry staining

The efficiency of delivery of recombinant AAV9 viral vector was assessed by fluorescence stating identifying the GFP carried by the vector. Hearts were harvested after the animals were sacrificed by CO_2_ asphyxiation. Trimmed cardiac tissue was embedded with optimal cutting temperature compound (OCT, Sakura) on ice and then sliced into 5-μm-thick cryostat slides. An inverted fluorescence microscope was used to observe the expression of GFP which was excited at 488 nm. Several cardiac tissue slides were incubated with anti-Sfrp1 antibody (Abcam, 1:200) at 4 °C for 8 h. After washing, the slides were incubated with secondary antibody at room temperature for 30 min. A DAB kit (Beyotime) was used to visualize the bounded antibodies. Captured images were further analyzed by software Image J (Ver1.36, NIH).

### In situ apoptosis detection

The myocardium apoptosis was detected by terminal transferase UTP nick end labeling (TUNEL). A commercially available TUNEL kit (Roche) was used to detect the apoptosis in the cardiac cryostat slides described above. The protocol was carried out according to the instruction provided by the manufacturer. Results were observed with an inverted fluorescence microscope.

### Western blotting

The minced cardiac tissue was homogenized on ice and further lysed by RIPA buffer system (Santa Cruz) supplemented with PMSF (Santa Cruz). Total protein and nuclear protein were extracted with Total Protein Extraction Kit (Beyotime) according to the protocol provided by the manufacturer. The protein sample concentration was determined with a BCA kit (Pierce). Protein samples were subjected to sodium dodecyl sulfate polyarylamide gel electrophoresis (SDS-PAGE) and then transferred to PVDF membranes. After incubated with QiuckBlock™ blocking buffer, the membranes were incubated with antibodies against β-catenin, Bax, Bcl-2, c-Myc, Sfrp1, and GAPDH respectively in 4 °C for 10 h. After washed by TBST, the membranes were further incubated with horseradish peroxidase- conjugated secondary antibodies at roome temperature for 30 min. Then the membranes were developed by the SuperSignal West Pico Chemilluminsecsence Substrate (Thermo) and subsequently visualized on X-ray films.

### Statistical analysis

Data collected in this study was presented in a (mean ± SD) manner and analyzed by SPSS statistical software (Ver16.0, SPSS). The differences between groups were analyzed by Student’s t-test and one-way ANOVA. NSK test was carried out as post-hoc tests. *P* < 0.05 was considered to indicate a statistically significant difference.

## Results

**Fig. 1:**
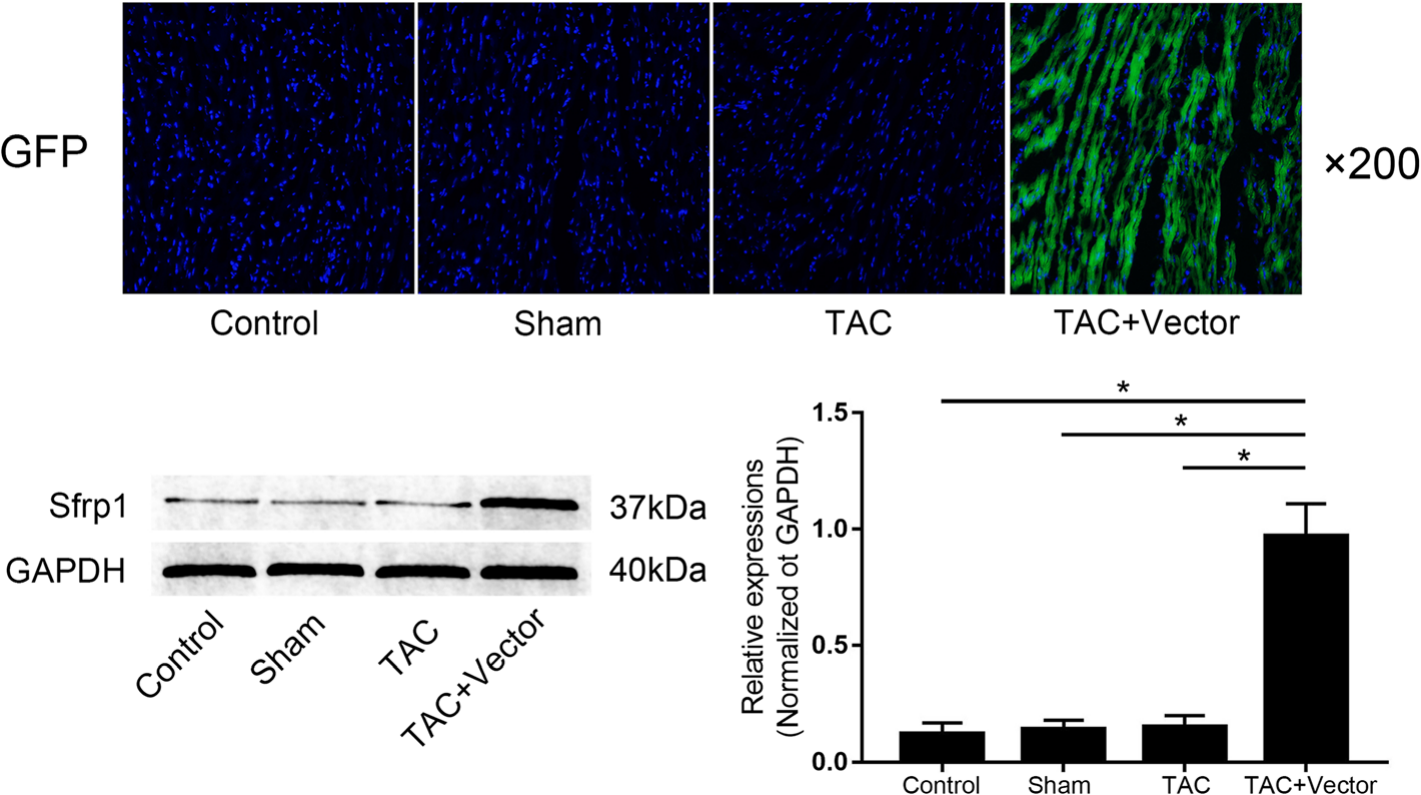
Images on the upper panel indicated the expression of GFP (green fluorescence) which indicated the distribution of AAV-Sfrp1 viral vector in myocardium from mice in control group, sham group, TAC group and TAC + Vector group. The left side of the lower panel showed the immunoblots of Sfrp1 in myocardium. Columns on the right side indicated the relative expression levels of Sfrp1 in myocardium from mice in control group, sham group, TAC group and TAC + Vector group respectively. [* differences were significant (*p* < 0.05)]

Sfrp1 was effectively up-regulated in myocardium received AAV9 viral vector delivery. The results were demonstrated in Fig. 1. One month after the delivery of AAV9, we found that the GFP carried by the viral vector was highly expressed in myocardium of mice. Moreover, compared with normal control animals, the expression level of Sfrp1 was significantly increased in mice received AAV9 viral vector delivered via tail vein.

**Fig. 2:**
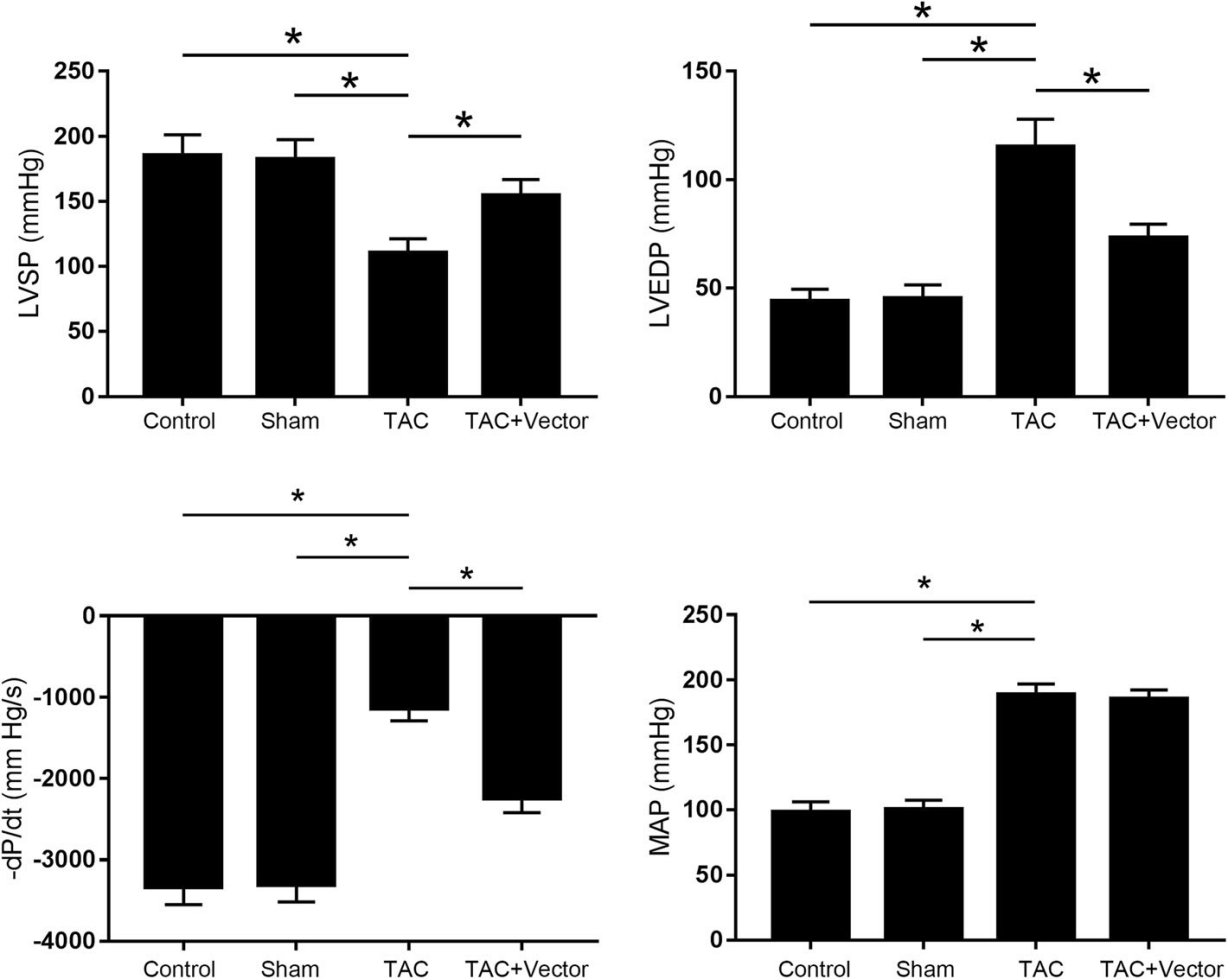
Columns on the left side and right side indicated the detected LVSP, LVEDP, MAP and –dP/dt in mice in control group, sham group, TAC group and TAC + Vector group respectively. [* differences were significant (*p* < 0.05)]

Sfrp1 viral vector administration improved cardiac function in TAC mice model. One month after viral vector administration, mice were subjected to TAC modeling. As shown in Fig. 2, the significantly increased MAP and impaired cardiac functions confirmed the successful establishment of TAC model. Compared with normal control, the LVSP decreased while the LVEDP increased in mice subjected to TAC. However, the LVSP was dramatically increased while LVEDP as well as –dP/dt were significantly reduced in Sfrp1 viral vector administrated mice exposed to TAC.

**Fig. 3:**
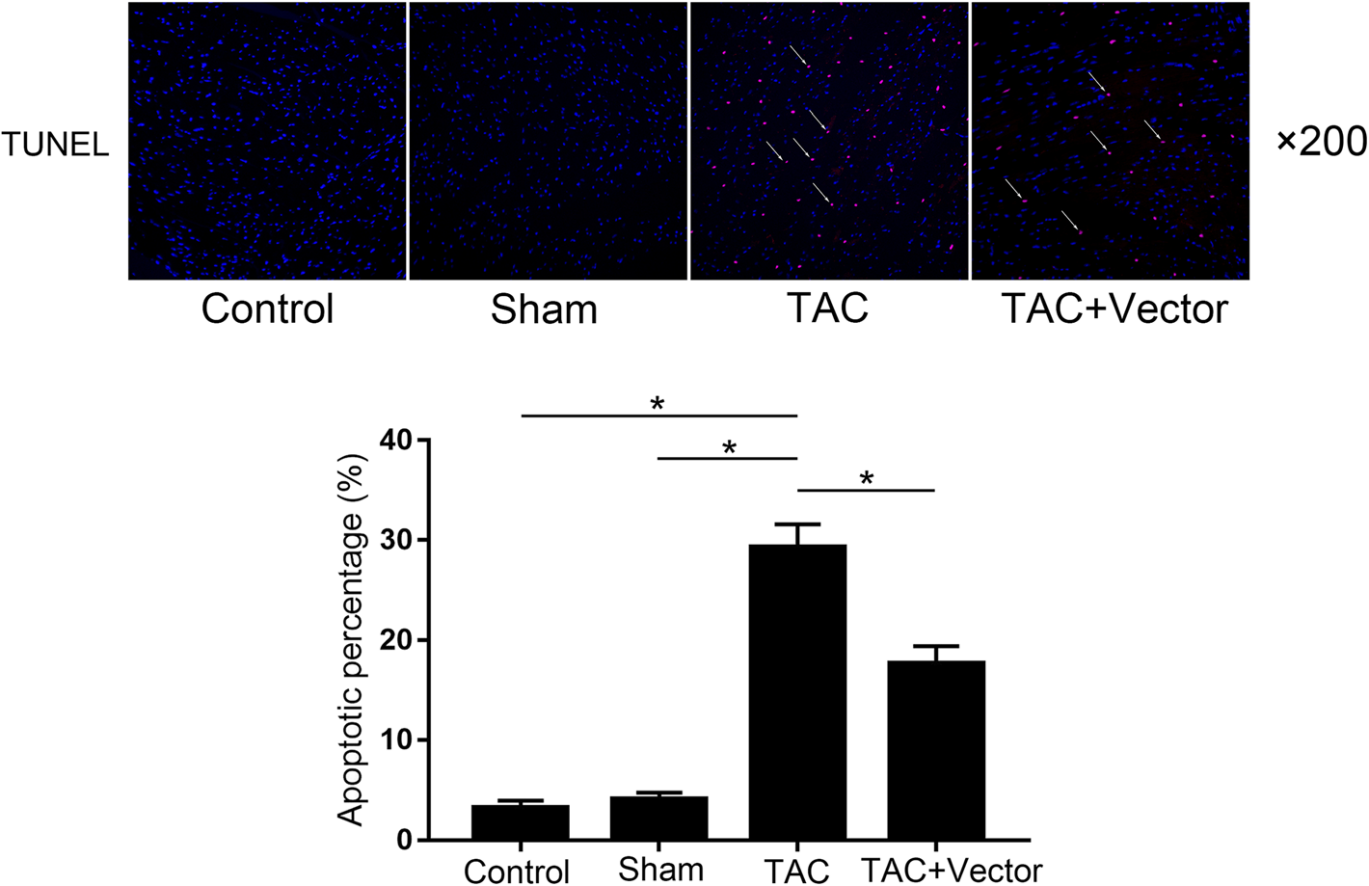
Images on the upper panel showed the results of TUNEL assay of myocardium. The white arrows are pointing at the TUNEL-positive cells. Columns on the lower panel indicated the apoptotic percentage detected in myocardium from mice in control group, sham group, TAC group and TAC + Vector group respectively. [* differences were significant (*p* < 0.05)]

Sfrp1 viral vector administration suppressed myocardial apoptosis in TAC mice model. The related results were demonstrated in Fig. 3. Compared with control animals, more TUNEL-positive cells were found in myocardium from mice exposed to TAC. However, in Sfrp1 viral vector treated animals, the number of TUNEL-positive myocytes was found dramatically reduced.

**Fig. 4:**
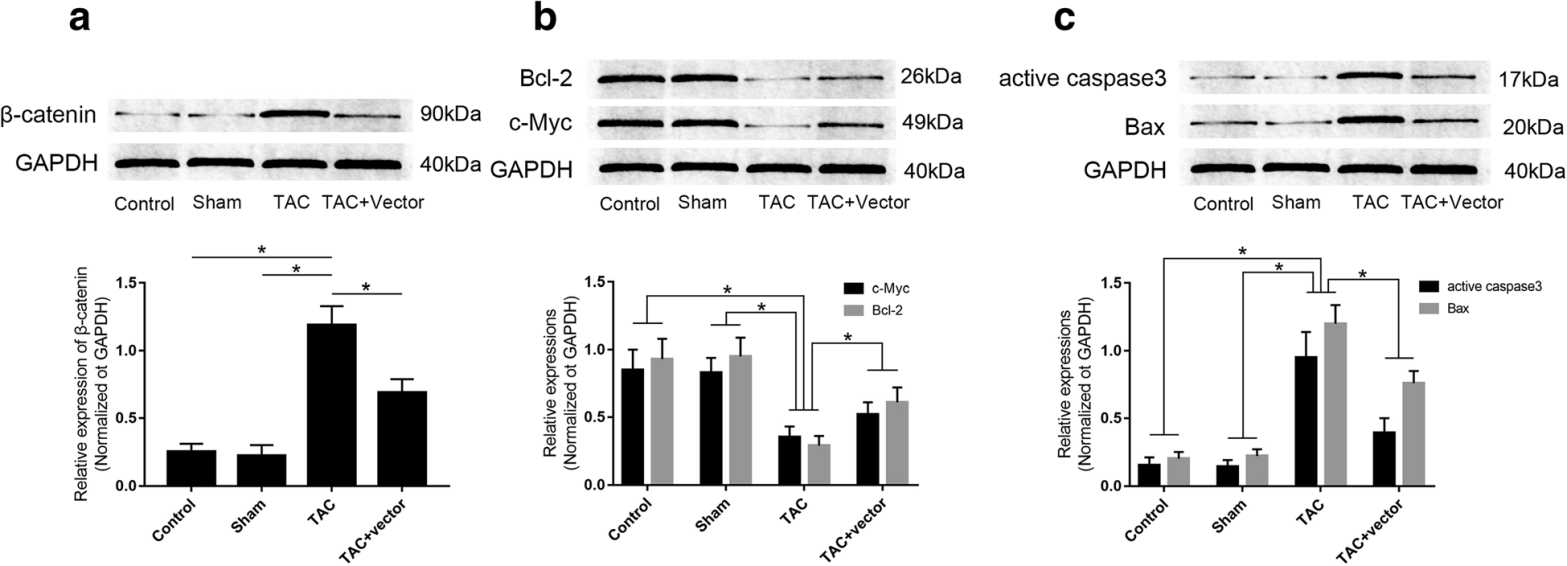
**a** the upper part demonstrated the immunoblots of β-catenin and GAPDH in myocardium. Columns on the lower panel indicated the relative expression level of β-catenin in myocardium from mice in control group, sham group, TAC group and TAC + Vector group respectively. **b** the upper part demonstrated the immunoblots of Bcl-2, c-Myc and GAPDH in myocardium. Columns on the lower panel indicated the relative expression level of Bcl-2 and c-Myc in myocardium from mice in control group, sham group, TAC group and TAC + Vector group respectively. **c** the upper part demonstrated the immunoblots of active caspase3, Bax and GAPDH in myocardium. Columns on the lower panel indicated the relative expression level of active caspase3 and Bax in myocardium from mice in control group, sham group, TAC group and TAC + Vector group respectively. [* differences were significant (*p* < 0.05)]

Sfrp1 viral vector administration inhibited activation of Wnt/β-catenin apoptotic signaling. The expression levels of key proteins of Wnt signaling were indicated by immunoblotting which was demonstrated in Fig. 4. The expression level of Sfrp1 was significantly up-regulated in mice received Sfrp1 viral vector administration. The expression level of β-catenin was found increased in myocardium exposed to TAC. As a result, the expression levels of active caspase3 as well as Bax increased while the expression levels of Bcl2 and c-Myc decreased in myocardium from TAC mice. The treatment of Sfrp1 viral vector, however, decreased the expression levels of active caspase3 and Bax but reduced the expression levels of Bcl2 and c-Myc in TAC- exposed myocardium.

## Discussion

Hemodynamic changes induced by hypertension, valvular and congenital heart diseases might lead to elevated cardiac workload. At the early stage, the heart attempts to maintain normal systolic function by mediating myocytes hypertrophy [11]. However, when the hemodynamic workload is prolonged, the compensatory capacity of the heart would be overwhelmed and further develop into heart failure which is recognized as the end-stage of various heart diseases causing heavy burden as a public health issue worldwide. It is well-accepted that the myocardium apoptosis is a fundamental mechanism of heart failure due to excessive loss of contractile units [12]. In this study, we used a TAC mice model to simulate the hemodynamic workload- induced heart failure. Evidenced by invasive hemodynamic examinations, both of the systolic and diastolic cardiac functions were significantly impaired in TAC mice model. Correspondingly, the myocardium apoptosis was found dramatically increased in hearts exposed to TAC procedure.

The Wnt signaling is a highly conservative pathway regulating various cellular biological processes. Under normal physiological conditions, Wnt signaling is static. However, in response to harmful stimuli induced by pathogens, the Wnt signaling would be activated and trigger multiple biological effects by directing expression of its down-stream genes [13]. According to several previous investigations, apoptosis was proved to be associated with the aberrant activation of Wnt signaling [14]. Conditional activation of Wnt signaling would lead to elevated expression of stable β-catenin which further participated in inducing apoptosis by regulating several pro-apoptotic such as caspase3 and anti-apoptotic genes such as bcl-2 [15, 16]. In the current study, we found that the expression level of β-catenin was increased significantly in myocardium from TAC mice. Accordingly, the expression levels of pro-apoptotic proteins, namely active caspase3 and Bax were found dramatically increased. The expression levels of the anti-apoptotic proteins, namely Bcl-2 and c-Myc were found suppressed in myocardium of TAC mice.

Sfrps are a set of proteins acting as regulators of Wnt signaling. They regulate Wnts by binding Wnts with their cysteine-rich domain which is structurally similar to the homologous region of the frizzled receptors [17]. This binding would sequester Wnts away from active receptor complexes and thus inhibits the activation of Wnt signaling pathway [18]. Several previous studies suggested the anti-apoptotic role of Sfrp on cells exposed to hypoxia, irradiation and inflammation [19, 20]. In one of our previous studies, we constructed a viral vector AAV9-Sfrp1 which was transfected into H9C2 myocytes. We found that Sfrp1 expression was elevated in targeted H9C2 cells and made these myocytes resistant to oxygen deprivation- induced apoptosis [9]. In the current study, the constructed AAV9-Sfrp1 viral vectors were delivered to mice with tail vein injections. Three weeks after delivery, the viral vectors were stably distributed and Sfrp1 was highly expressed in myocardium. Notably, evidenced by invasive hemodynamic assessments, both of the systolic and diastolic cardiac functions were preserved in TAC mice administrated with AAV9-Sfrp1 viral vectors which significantly inhibited the myocardial apoptosis. Moreover, the expression levels of β-catenin, active caspase3 and Bax were reduced while the expression levels of Bcl-2 and c-Myc were increased in AAV9-Sfrp1 viral vector- administrated TAC mice.

## Conclusions

In summary, our data from this study proved the role of Wnt signaling in TAC- induced cardiac dysfunction. Specifically, Wnt/β-catenin signaling pathway was activated which further mediated myocardial apoptosis by regulating expression of pro-apoptotic and anti-apoptotic genes. The AAV9-Sfrp1 viral vector was successfully delivered into myocardium and effectively up-regulated Sfrp1 expression which is an inhibitor of Wnt signaling. As a result, the AAV9-Sfrp1 viral vector improved cardiac systolic and diastolic functions via inhibiting Wnt pro-apoptotic signaling in myocardium from TAC mice.

## Abbreviations

AAV9: Adeno-associated virus 9
-dP/dt: Maximum rate of left ventricular pressure decay
LVSP: Left ventricular systolic pressure
MAP: Mean arterial pressure
Sfrp1: Secreted frizzled related protein 1
TAC: Transverse aortic constriction
